# Pre-Implantation Genetic Testing for Monogenic Disorders (PGT-M) in A Family with A Novel Mutation in *DPAGT1* Gene 

**DOI:** 10.22074/cellj.2021.7335

**Published:** 2021-10-30

**Authors:** Zahra Tabatabaei, Khadijeh Karbalaie, Parham Habibzadeh, Mohammad Ali Farazi Fard, Mohammad Ali Faghihi, Mohammad-Hossein Nasr Esfahani

**Affiliations:** 1. Persian BayanGene Research and Training Center, Shiraz University of Medical Sciences, Shiraz, Iran; 2. Department of Animal Biotechnology, Cell Science Research Center, Royan Institute for Biotechnology, ACECR, Isfahan, Iran; 3. Center for Therapeutic Innovation, Department of Psychiatry and Behavioral Sciences, University of Miami Miller School of Medicine, Miami, USA; 4. Isfahan Fertility and Infertility Center, Isfahan, Iran

**Keywords:** Congenital Disorders of Glycosylation, Genetic Testing, *In Vitro* Fertilization, Next Generation Sequencing

## Abstract

Congenital disorders of glycosylation (CDG) are a heterogeneous group of systemic disorders characterized by defects in glycosylation of lipids and proteins. One of the rare subtypes of CDG is CDG-Ij (MIM # 608093), which is caused by pathogenic mutations in *DPAGT1*, a gene encoding UDP-N-acetylglucosaminedolichyl-phosphate N-acetylglucosaminephosphotransferase enzyme. This enzyme catalyzes the first step of oligosaccharide synthesis in glycoprotein biosynthesis pathway. Preimplantation genetic testing for monogenic disorders (PGT-M) is a diagnostic technique that can reveal the genetic profile of embryos before implantation phase of *in vitro* fertilization (IVF). Currently, this approach is performed using next generation sequencing (NGS) technology. Herein, with the help of whole-exome and Sanger sequencing, we detected a novel missense mutation (NM_001382, c.1217 A>G) in *DPAGT1* gene in two families with consanguineous marriage. Using different online bioinformatics tools including MutationTaster, I-Mutant v2.0, T- Coffee, and CADD v1.0, this mutation was predicted pathogen. Finally, after performing PGT-M followed by successful pregnancy, a normal child was born in one of these families. In conclusion, we identified a novel pathogenic mutation in *DPAGT1* in a family with multiple members affected by CDG, which extends the range of pathogenic variants associated with CDG and therefore facilitates early detection of the disease.

## Introduction

Lipid or protein glycosylation defects result in a heterogeneous group of neurometabolic disorders that are collectively called “congenital disorders of glycosylation” (CDG) (1). N-glycosylation and O-glycosylation are the two major types of glycosylation. Defective N-glycosylation results in type I and II CDG (2, 3). These two conditions are differentiated based on the underlying pathophysiology; the former is mainly due to glycan biosynthesis errors, while the latter results from errors in processing of the produced glycan. DPAGT1-CDG (CDG-Ij, MIM # 608093) is a rare autosomal recessive disorder caused by pathogenic mutations in *DPAGT1*, encoding UDP_ N- acetylglucosamine- dolichyl-phosphate N- acetylglucosaminephosphotransferarse (GPT), the enzyme taking part in dolichol-linked oligosaccharide pathway (4, 5). The DPAGT1-CDG-Ij disease is characterized by failure to thrive and feeding difficulties that become evident soon after the birth. Moreover, neurological signs, including tremor, clonus, and muscle fasciculation may soon be seen. Neurological abnormalities, including: psychomotor retardation, seizures, mental retardation, hyperexcitabilty, ataxia, and progressive microcephaly may appear. In addition, liver dysfunction in these individuals can lead to coagulopathy and hypoproteinemia. Some individuals with CDG have facial anomalies, inverted nipples, and subcutaneous fat pads (6-9).

Over the last four decades, assisted reproductive technologies (ART), including* in vitro* fertilization (IVF) and intra-cytoplasmic sperm insemination (ICSI), have led to the birth of over five million children (10). Recent advances in this field have brought hope for couples who have had children afflicted with different genetic disorders. In the past, preimplantation genetic testing for monogenic disorders (PGT-M) was performed using fluorescent in situ hybridization (FISH) and comparative genomic hybridization (CGH) for screening embryos with aneuploidy or chromosomal rearrangement. However, recently more comprehensive and advanced genetic diagnostic techniques such as whole-exome sequencing (WES) have supplanted the aforementioned techniques. 

To date, there are only a few reports of patients with CDG due to pathogenic mutations in *DPAGT1*. Identification of various mutations responsible for the development of the disease bolsters diagnostic precision and subsequently paves the way for better genetic counseling and PGT-M. In this study, we report on a family with multiple individuals affected by CDG due to a novel mutation in *DPAGT1* gene and subsequently present the outcome of the PGT-M based on the identified variant for one of these couples.

### Cases report

**Fig.1:**
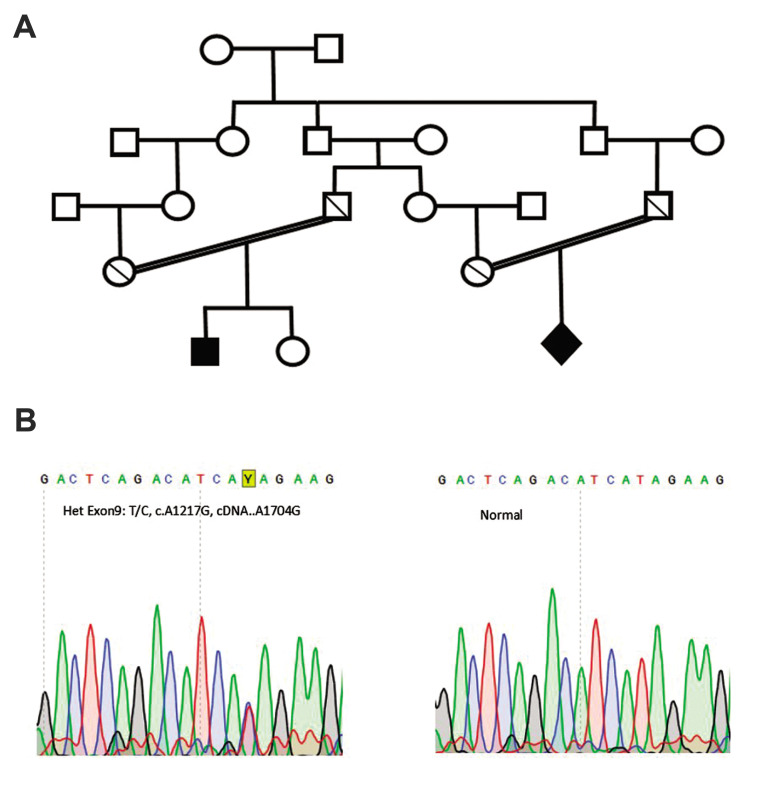
Family pedigree and sequencing chromatograms of two families. **A.** Pedigree and kinship relationship of the apparently normal couples. The first couple (left side) had a deceased 4-month boy (proband) and requested PDG for their second pregnancy. The second couple (right side) had an aborted embryo. **B.** Sequencing chromatograms of both couples show heterozygous mutation for the identified variant. **C.** The normal sequencing chromatogram of the unaffected girl embryo which was homozygous for the wild type variant.

All couples provided written informed consent for clinical and molecular studies. The Ethics Committee of the Persian BayanGene Research and Training Center approved the protocol (PBG- 06122016- 5708). As illustrated in Figure 1, our participants are couples from a big family.

The proband was a product of consanguineous marriage who was born with low Apgar scores. He was subsequently admitted to neonatal intensive care unit (NICU). He had severe hyperexcitability, hypotonia and swallowing difficulty. A thorough laboratory investigation including chromosomal analysis and metabolic evaluation was performed. However, no abnormality was identified. Brain computed tomography (CT) revealed no structural abnormalities. Electroencephalography and echocardiography were normal. The patient was discharged from the hospital. However, he was readmitted after a week due to severe respiratory distress. Clinical and imaging evaluations were in favor of aspiration pneumonia. The patient was subsequently intubated and placed on mechanical ventilation. Broad-spectrum antibiotics were started. However, his clinical condition deteriorated and he subsequently died at the age of four months. The parents of the affected individual and another couple in the family with an aborted fetus were subsequently referred for genetic counselling. 

Genomic DNA was isolated from blood samples of both couples and the aborted fetus using QIAamp DNA Mini kit (Qiagen, Germany). The optical density of the extracted DNA was examined at 260 nm and 280 nm using the Nanodrop Analyzer (ND-1000) spectrophotometer (Thermo Fisher Scientific, USA).

The extracted DNA was used for WES using Illumina HiSeq2000 platform, by a standard Illumina protocol for pair-end 99-nucleotide sequencing. Sequencing was performed to sequence close to 100 million reads. The results of WES were aligned using BWA aligner tool (11). Consequently, the variants were identified and annotated via GATK (12) and ANNOVAR (13) software programs. A novel homozygous missense mutation in exon 9 of DPAGT1 gene (NM_001382: c.A1217G:p.Y406C) was identified in the aborted fetus. Sanger sequencing was subsequently used to study the identified mutation in other family members. The following primers were used to amplify the exon 9 of DPAGT1 gene and the flanking intronic sequences:

F: 5ˊ-CTGAAATGTGAGTGTGGATAAC-3ˊ

R: 5ˊ-CCATACATGAGAGAAACCTC-3ˊ

Results of sequencing were analyzed using 4-peaks software, which confirmed that both couples were heterozygous and that the aborted fetus was homozygous for the identified mutation (Fig .1). The detected mutation was analyzed with MutationTaster, a mutation predictor software for deep-sequencing (14), and I-Mutant v2.0, for the prediction of the protein stability upon mutations (15). Both of these softwares are free web-based applications. Furthermore, the amino acid sequence of GPT in different species was aligned with T-Coffee, a multiple sequence alignment software (16). This mutation was also analyzed using published Combined Annotation Dependent Depletion software (CADD v1.0), a web-based application that scores any possible human single-nucleotide variant (SNV) (17). Analysis using MutationTaster, blastn and blastp showed that the amino acid under investigation was conserved in all available sequences of mammalian species (Table 1). The reliability index (RI) of this protein was found to decrease upon tyrosine substitution, measured by protein sequence analysis with I-Mutant v2.0. In addition, this software indicated that substitution of this amino acid with any other amino acids caused a decrement in RI (Table 2A). Sequence alignment of this protein with T-Coffee showed that this amino acid was conserved (Table 2A). This mutation had a raw score of 3.96 and a Phred-like score (C-score) of 20.3 when analyzed with the CADD program as a single-nucleotide variation (SNV).

**Table 1:** The evolutionary conservation for *DPAGT1* in other species at the amino acid level based on MutationTaster analysis. These homologus sequences were aligned with the corresponding human *DPAGT1* sequence using **A.** Blastp and **B.** Blastn respectively


Species	Match	Gene	NT	Alignment

A				
Human			406	F S I R Y Q L V R L F Y D V
Mutated	Not conserved		406	F S I R Y Q L V R L F C D V
Chimpanzee	All identical	ENSPTRG00000004370	406	F S I R Y Q L V R L F Y D V
Mmulatta	All identical	ENSMMUG0000014872	406	F S I R Y Q L V R L F Y D V
Fcatus	All identical	ENSFCAG00000001163	406	F S I R Y Q L V R L F Y D V
Mmusculus	All identical	ENSMUSG00000032123	408	F S I R Y Q L V R L F Y D V
Ggallus	All identical	ENSGALG00000000285	407	F S I R Y Q L V R L F Y D V
Trubripes	All identical	ENSTRUG0000001661	414	F S I R Y Q L V R L F Y D V
Drerio	All identical	ENSDARG00000061061	404	F S I R Y Q L V R L F Y D V
Dmelanogaster	All identical	FBgn0032477	406	F S I R Y Q L V R L F Y
Celegans	All identical	Y60A3A.14	410	F S I R Y Q L V R L F Y D V
Xtropical	All identical	ENSXETG00000021574	406	F S I R Y Q L V R L F Y D V
B				
Human		11324	11324	T C G A C T CT T C T A T G A T G T C T G A G T
Mutated	Not conserved	11324	11324	t c g a c t c t t c t g t g a t g t c t g a g
Ptroglodytes	All identical	7050	7050	t c g a c t c t t c t a t g a t g t c t g a g
Mmulatta	All identical	13780	13780	t c g a c t c t t c t a t g a t g t c t g a g
Fcatus	All identical	6806	6806	t c g a c t c t t c t a c
Mmusculus	All identical	8268	8268	t c g a c t c t t c t a t g a t g t c t g a g
Ggallus	No alignment	n/ a	n/ a	
Trubripes	No alignment	n/ a	n/ a	
Drerio	No alignment	n/ a	n/ a	
Dmelanogaster	No alignment	n/ a	n/ a	
Celegans	No alignment	n/ a	n/ a	
Xtropical	No alignment	n/ a	n/ a	


NT; Amino acid position and n/a; Not available.

**Table 2:** Sequence alignment of GPT protein.** A.** The measured level of protein stability, the change value of free energy (DDG) and the RI of GPT protein upon every single-point mutation in tyrosine position at 25˚C and neutral environment using I-Mutant v2.0. **B.** Multiple sequence alignment of GPT protein with T-Coffee showed that tyrosine 406 (Y) was a highly conserved residue during the evolution across the Mamalia class


A								
Position	WT	NEW	DDG	Stability	RI	pH	T	

406	Y	C	-0.02	Decrease	5	7	25	
406	Y	G	-3.58	Decrease	9	7	25	
406	Y	H	-1.67	Decrease	8	7	25	
406	Y	I	-0.30	Decrease	4	7	25	
406	Y	L	-0.26	Decrease	5	7	25	
406	Y	M	-0.51	Decrease	5	7	25	
406	Y	T	-0.88	Decrease	7	7	25	
406	Y	P	-1.26	Decrease	3	7	25	
406	Y	S	-1.64	Decrease	8	7	25	
406	Y	A	-1.94	Decrease	9	7	25	
406	Y	V	0.16	Decrease	2	7	25	
406	Y	K	-0.26	Decrease	5	7	25	
B																														
Species	Protein	Alignment																												

Homo sapiens	NP_001373.2	T	L	L	L	L	L	L	Q	I	L	G	S	A	I	T	F	S	I	R	Y	O	L	V	R	L	F	**Y**	D	V
Mus musculus	NP_031901.2	T	L	L	L	L	L	L	Q	V	L	S	S	A	A	T	F	S	I	R	Y	Q	L	V	R	L	F	**Y**	D	V
Rattus norvegicus	NP_955420.1	T	L	F	L	L	L	L	Q	V	L	S	S	A	V	T	F	S	I	R	Y	Q	L	V	R	L	F	**Y**	D	V
Danio rerio	NP_001082880.1	T	A	I	M	L	L	M	Q	V	L	G	S	A	V	A	F	G	I	R	Y	H	L	V	R	L	F	**Y**	D	V
Cricetulus griseus	NP_001230970.1	T	L	L	L	L	L	L	Q	I	L	S	S	A	V	T	F	S	I	R	Y	Q	L	V	R	L	F	**Y**	D	V
Macaca mulatta	NP_001244785.1	T	L	L	L	L	L	L	Q	I	L	G	S	A	F	T	F	S	I	R	Y	Q	L	V	R	L	F	**Y**	D	V
		*		:	:	*	*	:	*	:	*	.	*	*		:	*	.	*	*	*	:	*	*	*	*	*	*	*	*


WT; Amino acid in wild type protein, NEW; n\New amino acid after mutation, DDG; DG (new protein)-DG (wild type) in kcal/mol, DDG <0; Decrease stability, DDG >0; Increase stability, RI; Reliability index, pH; -log [H+], T; Temperature (°C), *; Identical amino acids, and :; Just identical amino acids.

Following verification of heterozygous mutation in the couple, they were referred to Isfahan Fertility and Infertility Center, Iran. Upon their request, intracytoplasmic sperm injection (ICSI) was carried out based on routine method performed in the center (18). Using the gonadotropin-releasing hormone (GnRH) agonist protocol, hyper ovulation was induced. In this aim 150 IU of Cinal-F (CinnaGene, Iran) and 75 Menogon (Ferring, Germany) were used. Monitoring was carried out with using vaginal ultrasound. Cetrotide (Merck-Serono, Germany) was administered when the size of the dominant follicle was 13 mm. Ovulation induction was induced with 10,000 IU of human chorionic gonadotropin (hCG, Ferring, Germany). Thirty-six hours post-hCG, the ovum was picked up and three mature meiosis 2 (MII) oocytes were obtained. ICSI was carried out according to the standard protocol. On day 3, two embryos in 8-16-cell stage were found suitable for blastomere biopsy. Each blastomere was washed in phosphate buffered saline without calcium and magnesium (PBS) and transferred to polymerase chain reaction (PCR) tube for DNA amplification using REPLI-g Single Cell kit (Qiagen, Germany). PCR was subsequently performed on the amplification products of the exon 9 of the *DPAGT1* gene. Sanger sequencing was then performed on the PCR products to determine the genotype of the embryos using 3130xl Genetic Analyzer (Applied Biosystems, USA). Based on the results of the Sanger sequencing, two blastomeres were found unaffected and one of them was transferred which resulted in the birth of a healthy girl.

## Discussion

Glycosylated proteins play an important role in many biological pathways including cell signaling, protein stability and immune defense (19). In this study, we identified a novel pathogenic missense mutation in *DPAGT1* gene leading to congenital disorder of glycosylation (CDG) in a family. The pathogenic nature of the identified variant was supported by the absence of this variant in our local database and other publicly available genetic databases. In addition, highly conserved nature of the amino acid affected by this mutation, and extensive analysis of other individuals in the family, reinforced the pathogenic nature of the detected variant. Furthermore, PGT-M based on the identified variant resulted in a normal healthy child.

All patients affected by CDG, but those who suffered from CDG-Ib, had similar clinical presentations with substantial variation in severity (20). Compared with previous reports, the patient reported in this study had the most severe clinical features, presenting with serious problems at birth that led to death by the age of four months. Pathogenic mutations in *DPAGT1* are also known to cause limb-girdle congenital myasthenic syndrome with tubular aggregates, highlighting the importance of N-glycosylation of proteins in maintaining the function of the neuromuscular junction (21). Since the patient in this report did not undergo a thorough clinical evaluation, this disorder could not be ruled out in this patient.

The rate of consanguineous marriage in any society depends on a wide range of social, religious, and demographic factors (22, 23). With a mean rate of 38.6%, in some community, Iran is one of the countries with the highest rates of consanguineous marriage (24). Therefore, autosomalrecessive disorders pose a major issue to this society. This underscores the importance of causative genetic variants identification in these disorders and subsequently, taking advantage of PGT-M techniques to prevent the transfer of deleterious variants to the next generation. 

## Conclusion

We identified a novel pathogenic mutation in *DPAGT1* gene based on bioinformatics analysis and genome sequencing. Subsequently, based on couples’ request, PGT-M was performed, which resulted in the birth of a healthy girl. 
